# Cross-border outbreak of *Yersinia enterocolitica* bioserotype 2/O:9 infections associated with consumption of French unpasteurised soft goat’s milk cheese, 2024

**DOI:** 10.2807/1560-7917.ES.2025.30.26.2500002

**Published:** 2025-07-03

**Authors:** Cyril Savin, Nathalie Fredriksen, Clémentine Calba, Lorraine Puzin, Arnaud Felten, Stéphane Larréché, Anthony Roche, Martine Denis, Anne-Sophie Le Guern, Fanny Chereau, Y. enterocolitica Outbreak Investigation Group, Yersinia enterocolitica Outbreak Investigation Group, Marie Briatte, Linda Ducret, Sophie Edouard, Lauranne Figuière, Stéphanie Giudicelli, Laura Guichard, Emmanuelle Houard, Syria Laperche, Edith Laurent, Carine Martins, Wesley Mattheus, Mohammed Umaer Naseer, Javier Pizarro-Cerdá, Elena Portell Buj, Catherine Ragimbeau, Manon Tasset, Sylvain Traynard, Telma Velez

**Affiliations:** 1 Institut Pasteur, Université Paris Cité, Yersinia Research Unit, Yersinia National Reference Laboratory, WHO Collaborating Research and Reference Centre for Plague FRA-146, Paris, France; 2 Mission des urgences sanitaires, Direction générale de l’alimentation, Paris, France; 3 French National Public Health Agency (Santé publique France), Provence-Alpes-Côte-d’Azur regional office, Marseille, France; 4 French Agency for Food, Environmental and Occupational Health and Safety (ANSES), Sequencing Platform (UGVB), Ploufragan-Plouzané-Niort Laboratory, Ploufragan, France; 5 Direction départementale en charge de la protection des populations (DDecPP) du Vaucluse, Avignon, France; 6 Direction départementale en charge de la protection des populations (DDecPP) des Alpes-de-Haute-Provence, Dignes-les-bains, France; 7 French Agency for Food, Environmental and Occupational Health and Safety (ANSES), Unit of Hygiene and Quality of Poultry and Pork Products (UHQPAP), Ploufragan-Plouzané-Niort Laboratory, Ploufragan, France; 8 French National Public Health Agency (Santé publique France), Department of Infectious Diseases, Saint-Maurice, France; 9 The members of the group are listed under Collaborators

**Keywords:** *Yersinia enterocolitica*, outbreak, cross-border, genomic surveillance, raw milk, goat cheese

## Abstract

In March 2024, the French genomic surveillance of enteric yersiniosis identified a cluster of lineage 2/3–9b *Yersinia enterocolitica* isolates, corresponding to bioserotype 2/O:9. An outbreak investigation was conducted to identify the source and implement control measures. A total of 175 confirmed cases were identified in France with sampling dates between 27 January and 23 August. Case interviews and trace-back investigations identified unpasteurised soft goat’s milk cheese from one manufacturer in France as the probable source of the outbreak. *Yersinia enterocolitica* belonging to the same cluster as the case isolates was isolated from 23 samples from the manufacturer and a goat farm supplying milk to the dairy. Cheeses from the manufacturer were recalled in France. A Rapid Alert System for Food and Feed (RASFF) notification was issued, allowing the identification of cheese distribution from the manufacturer to 29 countries. An Epipulse alert led to the identification of seven additional cases in Belgium, Luxembourg and Norway, illustrating the value of international warning systems. This outbreak demonstrates risks with consumption of unpasteurised cheese and emphasises the need of rigorous good hygiene practices on dairy farms and dairies, especially when milk is processed without pasteurisation.

**Key public health message**

**What did you want to address in this study and why?**

*Yersinia enterocolitica* is one of the most common causes of bacterial diarrhoea in Europe. People usually get infected by eating contaminated food, such as undercooked pork meat or raw vegetables. An outbreak of *Y. enterocolitica* occurred in France in 2024. This investigation aimed to find the source of the infections and prevent further cases.

**What have we learnt from this study?**

This large outbreak of *Yersinia enterocolitica* infections affected at least 175 people in France and seven in three other European countries. The likely source of the outbreak was consumption of contaminated soft unpasteurised goat's milk cheese. We detected *Y. enterocolitica* in samples from the cheese manufacturer and a milk producer. These isolates were genetically close to isolates from the diseased individuals.

**What are the implications of your findings for public health?**

Consumption of unpasteurised milk products can be a source of *Y. enterocolitica* and lead to large outbreaks. Good hygiene practices during milking, such as washing the udders of goats, could prevent contamination of milk. It is also important to better understand the circulation of pathogenic bacteria in ruminant herds, contamination routes of milk and persistence of pathogenic bacteria in cheese.

## Background

Surveillance of foodborne and waterborne diseases has been improved in recent years by the introduction of affordable and high-throughput genome sequencing [1]. Numerous genome-based methods have been developed to identify clusters of closely related bacterial isolates. Core genome multilocus sequence typing (cgMLST) schemes have proven to be powerful tools with high discriminatory power and do not require bioinformatics skills [2,3]. Genomic surveillance enables identification of a genetic link between isolates, which allows the public health authorities to quickly initiate an outbreak investigation, potentially leading to identification of a common source of exposure and implementation of control measures.

Enteric yersiniosis, caused by enteropathogenic *Yersinia*, is a gastroenteritis presenting with diarrhoea, abdominal pain and fever, and is predominantly diagnosed in children. Severe forms and invasive infections may be seen in older adults or patients with underlying conditions [4,5]. Yersiniosis is the third most commonly reported zoonosis in the European Union (EU) (7,919 confirmed cases in 2022) and is mainly caused by *Yersinia enterocolitica* [6]. In 2022, the incidence of enteric yersiniosis in Europe was estimated at 2.2 cases per 100,000 population. Notification is mandatory in 22 EU countries and voluntary in four (Belgium, France, Greece and Italy) [6]. Since 2016, France has annually reported the second highest number of cases (735–1,568, mean: 1,116) in Europe after Germany (1,814–2,763, mean: 2,152) [6]. Worldwide, most cases are considered sporadic, but outbreaks have been described and associated with consumption of pork products, vegetables or dairy products [7-9], even though trace-back investigations sometimes fail to identify the source of the contamination [10].

As both pathogenic and non-pathogenic lineages of *Y. enterocolitica* have been described, the pathogenic potential of the obtained isolates needs to be characterised [11]. In France, the *Yersinia* National Reference Laboratory (YNRL) conducts microbiological and molecular surveillance of *Yersinia* isolates from humans. Although the notification of enteric yersiniosis is not mandatory in France, *Yersinia* isolates are regularly sent on a voluntary basis by clinical microbiology laboratories from across the country to the YNRL for characterisation. Since 2018, genomes of *Yersinia* isolates are routinely sequenced, and species, lineage and pathogenic potential are identified using a 500-gene cgMLST *Yersinia* [11]. In France, enteric yersiniosis is mainly caused by *Y. enterocolitica* with the most common circulating subgroups being lineage 4 (87%), corresponding to bioserotype 4/O:3, followed by lineage 2/3–9b (11%), corresponding to bioserotype 2/O:9 [2]. Molecular typing of *Y. enterocolitica* is performed using a 1,727-gene cgMLST [2]. Clusters of genetically close isolates are notified to Santé publique France, the French National Public Health Agency, for assessment and epidemiological investigation.

## Outbreak detection

On 14 March 2024, the YNRL notified Santé publique France about a genomic cluster (number 916) consisting of six isolates of *Y. enterocolitica* lineage 2/3–9b, corresponding to bioserotype 2/O:9. These isolates were from five patients with sampling dates between 27 January and 2 March 2024. On 29 April, as the number of new cases identified weekly in the cluster had increased, an outbreak investigation was triggered.

The outbreak control team consisted of key actors of the French public health: the YNRL, Santé publique France, the French Agency for Food, Environmental and Occupational Health and Safety (ANSES) and the French General Directorate of Food (DGAL).

Here, we describe the outbreak investigation, with cases also in other European countries. We collaborated with authorities from other countries and aimed to assess the extent of the outbreak, identify its source and initiate appropriate control measures to prevent further cases.

## Methods

### Epidemiological investigation

#### Case definition and case finding

A French case was defined as an individual residing in France with laboratory-confirmed *Y. enterocolitica* infection since January 2024, with an isolate from faeces or blood characterised as lineage 2/3–9b, belonging to genomic cluster 916. French cases were identified by the routine microbiological surveillance conducted by the YNRL on *Yersinia* isolates sent on a voluntary basis by the French clinical microbiology laboratories.

A cross-border case was defined as a laboratory-confirmed case residing in a European country other than France, with an isolate sequenced according to the cgMLST scheme used for surveillance in the country of residence and belonging to the same genomic cluster 916. Cross-border cases were identified from European countries through Epipulse (the European surveillance portal for infectious diseases, run by the European Centre for Disease Prevention and Control (ECDC)).

#### Data collection and analysis

Demographics (age, sex (as binary variable), department of residence) of French cases were available from the clinical microbiology laboratories. All French confirmed cases or their legal representatives, if aged < 18 years, notified to Santé publique France between 29 April and 5 July were contacted for interviews by phone. Information on the type of symptoms, onset date and exposures were collected using a trawling questionnaire and iterative interviewing. Exposures included environmental exposures (swimming, farm visit and others), drinking water supply and food consumption (meat products (raw, cured, cold cuts), dairy products, raw and unpeeled vegetables and fruits) in the 7 days before the onset of symptoms. For each food item consumed, details on the brand, places and dates of purchase were collected. More precisely, a trawling questionnaire was used for the first 21 cases interviewed. For the following interviews, the questionnaire was expanded to include additional dairy products mentioned by several of the 21 cases. All interviewed cases were asked if they used a supermarket loyalty card and if so, their consent for transmission of the loyalty card number to the authorities responsible for trace-back investigations.

From the cross-border cases, we received information on sampling date, age, sex and country of residence. Details on travels and consumption of dairy products, if collected by the public health authorities in the country of residence, were also shared with the French public health authorities.

### Trace-back investigation

Food chain investigations were conducted by the competent authority (DDecPP) in the Provence-Alpes-Côte d’Azur region and by the DGAL for national and international investigations. The DGAL requested information on food purchases from the retailers where the cases had a loyalty card. The data were limited to dairy products purchased within the 2 months preceding the onset of symptoms. If the suspected type of dairy product was purchased, further trace-back data were obtained from the supermarkets to identify the manufacturer and batch numbers.

### Food and environmental sampling

Food and surface samples were taken at the implicated manufacturer. On each farm providing milk to the dairy, a pair of boot swabs was collected by walking through the entire area of the barn, and milk filters were sampled. All samples were sent to the ANSES for detection of *Y. enterocolitica.*

### Microbiological investigation

#### Specimens from French cases

For the French patients presenting with gastrointestinal symptoms, the clinical microbiology laboratories directly plated faecal specimens onto semi-selective cefsulodin-irgasan-novobiocin (CIN) agar media. For bacteraemia-suspected patients, blood specimens were cultured. Presumptive isolates of *Y. enterocolitica* were sent to the YNRL for confirmation and further characterisation.

#### Food and environmental samples

Detection of *Y. enterocolitica* was performed according to the International Organization for Standardization (ISO) method 10273:2017 [12]. Briefly, 25 g of food samples were diluted in 225 mL PSB (peptone sorbitol bile salts) broth, and the environmental swabs, milk filters and boot swabs were placed in 150, 250 and 250 mL of PSB broth, respectively. After homogenisation for 2 min, 10 mL of the broth was transferred to 90 mL ITC (irgasan-ticarcillin-chlorate) broth. The two broths, PSB and ITC, were then incubated at 25°C for 48 h. Thereafter, as described in the ISO method, the enrichment broth ITC was treated with KOH and a loopful of the enriched broths of PSB and KOH-treated ITC was streaked onto CIN plates and incubated at 28°C for 48 h. Typical red bull’s eyes colonies on CIN plates were streaked onto YeCM (*Y. enterocolitica* chromogenic medium, CHROMagar, Saint-Denis, France) plates [13] and incubated at 28°C for 24 h. Blue or green colonies were discarded, and only pink colonies, suspected to be pathogenic *Y. enterocolitica*, were confirmed as *Y. enterocolitica* by matrix-assisted laser desorption/ionisation time-of-flight (MALDI-TOF) (Bruker, Billerica, the United States (US)) and then biotyped according to the biogrouping scheme of Wauters [14].

#### Genomic characterisation and comparison of isolates

Extraction of DNA from the clinical isolates sent to the YNRL was performed on Maxwell CSC 48 (Promega, Madison, US) using Maxwell RSC Cultured Cells DNA kit (reference AS1620, Promega). Sequencing and identification of the species using a 500-gene cgMLST *Yersinia* was performed as described by Savin et al. [11]. Molecular typing of the isolates was performed as described by Le Guern et al. [2] using a 1,727-gene cgMLST scheme specific to *Y. enterocolitica* created in the Bacterial Isolate Genome Sequence Database (BIGSdb) software [15] in the Institut Pasteur’s MLST and cgMLST resources (https://bigsdb.pasteur.fr/yersinia). Clustering of the isolates was recorded with a three-mismatch allelic distance (AD) threshold.

In the ANSES, DNA was extracted from the food and environmental isolates with the QiaAmp DNA Mini kit (Qiagen, Hilden, Germany). Sequencing libraries were prepared using Illumina DNA library preparation kit, and 2 × 150 bp paired-end reads were sequenced on Illumina Nextseq2000 sequencer (Illumina, San Diego, US). The raw sequences were transferred to the YNRL for genome comparison.

## Results

### Epidemiological investigation

#### Description of the outbreak in France

**Figure 1:**
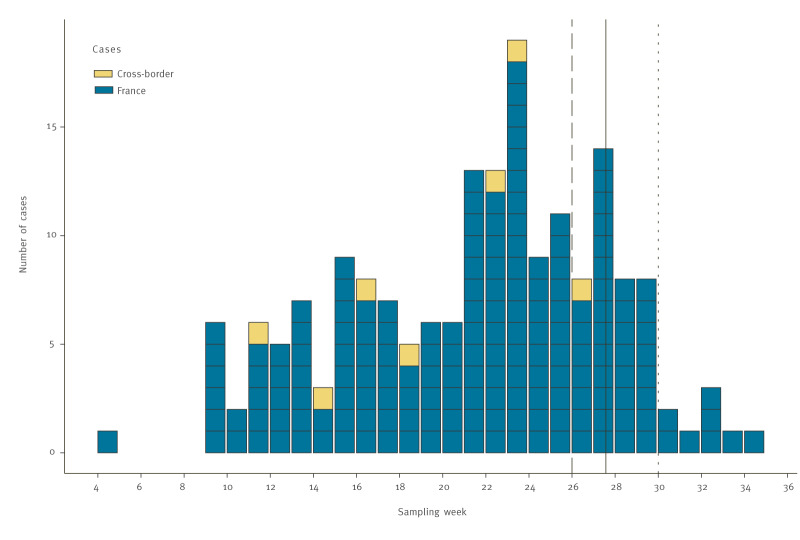
Timeline of confirmed cases of *Yersinia enterocolitica* lineage 2/3–9b (bioserotype 2/O:9) and control measures in an outbreak linked to consumption of unpasteurised soft goat’s milk cheese, by sampling week, France (n = 175) and other countries (n = 7), January–August 2024

**Figure 2:**
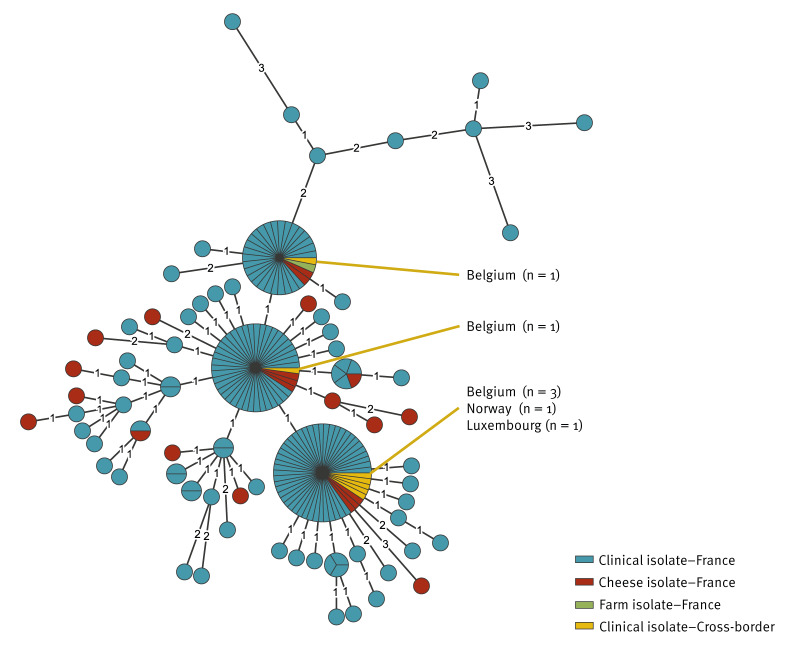
Minimum spanning tree of *Yersinia enterocolitica* lineage 2/3–9b, cluster 916 isolates from cases (n = 182), unpasteurised soft goat’s milk cheese (n = 22) and farm environment (n = 1), France (n = 198) and other countries (n = 7), January–August 2024

**Figure 3:**
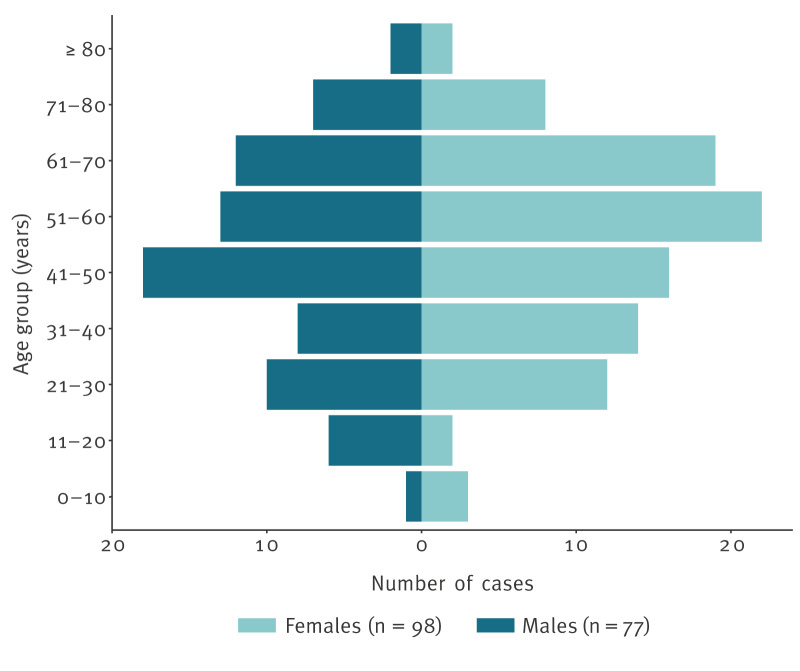
Age and sex distribution of confirmed cases of *Yersinia enterocolitica* lineage 2/3–9b bioserotype 2/O:9 cluster 916 in an outbreak linked to consumption of unpasteurised soft goat’s milk cheese, France, January–August 2024 (n = 175)

**Figure 4:**
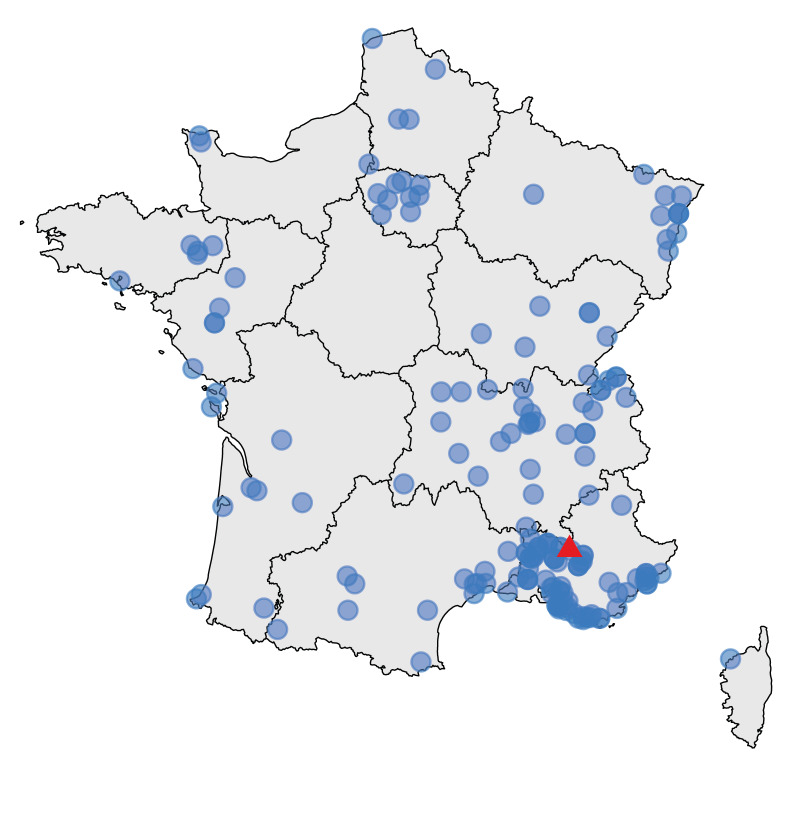
Geographical distribution of confirmed cases of *Yersinia enterocolitica* lineage 2/3–9b (bioserotype 2/O:9) cluster 916 in an outbreak linked to consumption of unpasteurised soft goat’s milk cheese, by postal code of residence, France, January–August 2024 (n = 175)

By 3 September 2024, 175 confirmed cases were identified by the YNRL with sampling dates between 27 January and 23 August (Figure 1). All isolates were identified as *Y. enterocolitica* lineage 2/3–9b, corresponding to bioserotype 2/O:9. The use of the *Y. enterocolitica* cgMLST with 1,727 genes followed by single linkage clustering with three-mismatch threshold [2] allowed us to group them into the same cluster 916 (Figure 2). Most (171/175) isolates were from faecal samples, four were from blood samples. The cases were aged 1–85 years (mean age: 48 years; interquartile range (IQR): 34–64 years), and 98 (56%) were females, 77 were (44%) males (Figure 3). These cases resided in 12 of the 13 regions of mainland France, however, 73 (42%) cases resided in the Provence-Alpes-Côte d’Azur region, southeast France (Figure 4).

The dashed vertical line marks the date of 24 June, the inspection of the cheese manufacturer. The solid vertical line marks the date of 5 July, the initiation of the recall of products from the cheese manufacturer. The dotted vertical line marks the date of 23 July, the initiation of the recall of products from farm A.

cgMLST: core genome multilocus sequence typing.

Numbers close to the branches correspond to the allelic distance between 2 nodes according to 1,727-gene cgMLST *Y. enterocolitica* [2].

Red triangle corresponds to the location of cheese manufacturer and the goat farm supplying milk from which isolates were isolated.

Santé publique France interviewed 57 (33%) French cases aged 3–85 years (mean age: 48 years; IQR: 37–61 years). Of these, 32 were females and 25 were males. The interviewed cases resided in 12 regions, 31 of them in Provence-Alpes-Côte d’Azur. The onset dates ranged from 10 March to 10 June. Most cases experienced abdominal pain (n = 50) and diarrhoea (n = 45), and 39 had both abdominal pain and diarrhoea. Other symptoms were fever (n = 25), nausea (n = 24), bloody diarrhoea (n = 10) and vomiting (n = 7). One case was hospitalised.

### Identification of the source of the outbreak

#### Food exposures

A preliminary analysis of the exposures of the first 21 interviewed cases did not suggest consumption of meat products as three cases never ate cold cuts and two never ate meat. Furthermore, four cases spontaneously mentioned consumption of an unpasteurised soft goat’s milk cheese from Provence-Alpes-Côte d’Azur region, a food item not included in the trawling questionnaire. Among the following 36 cases interviewed, with the question of this cheese included, 22 mentioned having consumed the cheese. No other food product was consumed by more than 50% of the interviewed cases.

#### Trace-back investigation

Of the 26 interviewed cases who had eaten the suspected unpasteurised soft goat’s milk cheese, 16 had purchased the cheese in a grocery store, six in a cheese shop, three either in a grocery store or in a cheese shop and one did not recall the place of purchase. Among the 19 cases with confirmed or possible purchases in grocery stores, 10 had loyalty cards. The DGAL identified purchases of this cheese for six cases from their loyalty cards and confirmed that all purchased cheeses came from one manufacturer. The batches and dates of purchase were listed for these six cases, and these persons had consumed cheese of different batch numbers.

#### Site inspections, food and environmental samples analysis

On 24 June, the DDecPP visited the manufacturer. The inspection did not reveal any hygiene deficiencies or non-conformities with regulations. Thirty-nine samples were taken from 30 cheeses, eight surface swabs and one other environmental sample. The ANSES detected *Y. enterocolitica* biotype 2 in four cheeses (22 isolates) and one environmental sample. The manufacturer received milk from eight dairy goat farms. These farms were inspected on 7–8 July and samples were taken from 30 milk filters and one pair of boot swabs per farm. *Yersinia enterocolitica* biotype 2 was isolated from a pair of boot swabs from one farm (Farm A), while all the other samples were negative. Farm A sold 65% of its milk production to the manufacturer and used 35% for its own cheese and yoghurt production.

The 23 cheese and environmental isolates were *Y. enterocolitica* 2/3–9b and belonged to cluster 916 (Figure 2). The 198 isolates from cases, cheese and environment were closely related to each other with a maximum AD of 3 between the two closest isolates. In addition, five groups of identical isolates (AD = 0) from cases and cheese were identified, and one group with 31 isolates included also the isolate from the pair of boot swabs of Farm A (Figure 2).

### Cross-border investigation

After issuing the European Commission’s Rapid Alert System for Food and Feed (RASFF) 2024.5201 notification on 9 July 2024, distribution of cheese from the manufacturer was identified in 17 EU countries and 12 other countries, including some in other continents. An EpiPulse event (2024-FWD-00058) was created on 9 July 2024, and whole genome sequencing (WGS) data of the first isolate found in cheese were shared. Seven cross-border cases were identified in three countries: Belgium (n = 5), Luxembourg (n = 1) and Norway (n = 1). Raw sequencing data reads from the seven isolates were sent to the YNRL who confirmed that they all were *Y. enterocolitica* lineage 2/3–9b belonging to cluster 916 (Figure 2).

The cross-border cases were sampled between March and June 2024. Food consumption or travel history could not be obtained for six cases and no goat’s milk cheese consumption or travel to France was reported for the seventh case.

## Outbreak control measures in France

A recall of all batches of unpasteurised soft goat’s milk cheese from the manufacturer was initiated on 5 July. Cheese production was interrupted and then resumed, after cleaning and disinfection, without using milk from Farm A. Sales were authorised after the ANSES conducted microbiological analyses and the results were negative.

After detection of *Y. enterocolitica* isolates closely related to case and cheese isolates in a sample from Farm A, the DDecPP visited Farm A on 22 July. A thorough cleaning of the farm and cheese-making room was done. Extensive investigations were conducted on the farm to understand the source of the contamination. No hygiene violations were identified, although the goats' udders were not cleaned before milking, as it is not mandatory. Milk was to be pasteurised until cleaning of goats' udders before milking was implemented, and microbiological analyses of milk filters and batches of raw goat's milk cheese showed no contamination with pathogenic *Y. enterocolitica*. A recall of all batches of cheeses made by Farm A was initiated on 23 July. Farm A did not export cheeses or milk products.

Authorisation to resume raw milk distribution and raw milk cheese production by Farm A was delivered by the DDecPP on 28 August.

Given the sepsis risk after blood transfusion associated with *Y. enterocolitica* [16], the French Blood Establishment (EFS) included a 2-month deferral period, from 21 June to 20 September 2024, for donors who reported having consumed the incriminated cheese in the 7 days before donation.

## Discussion

We describe here a large cross-border outbreak of *Y. enterocolitica* 2/O:9 involving 175 confirmed cases in France and seven in other European countries (five in Belgium, one in Norway and one in Luxembourg). For the first time in France, we were able to identify and microbiologically confirm the source of a *Y. enterocolitica* outbreak.

In France, although the cases were residing across the country, there was a southeast concentration, suggesting that the source could be a regional food specialty with a nationwide distribution. Iterative interviewing using trawling questionnaires identified an unpasteurised soft goat’s milk cheese produced in southeastern France as the probable source of the outbreak. This hypothesis was reinforced by trace-back investigations identifying a single cheese manufacturer and further microbiologically confirmed by detection of *Y. enterocolitica* closely related to the case isolates. Trace-back investigations identified eight milk suppliers of the cheese manufacturer, and Farm A was identified as the likely source of contamination with the isolation of *Y. enterocolitica* closely related to case and cheese isolates. The high number of cases identified through systematic genome sequencing of *Y. enterocolitica* isolates at the YNRL and cases interviewed by Santé publique France, allowed for early identification of a food item unlisted in the questionnaires, but spontaneously mentioned by only a few cases which might have otherwise gone unnoticed. The control measures implemented in July 2024 proved to be effective, with a sharp decrease in notified cases after the implementation and no cases detected after the end of August 2024.

Outbreaks of *Y. enterocolitica* infections associated with milk have been described in the US where the causative agent was *Y. enterocolitica* 1B/O:8 [8,17,18]. In Europe, 2018–2021, milk and milk products was the food category most frequently contaminated with *Y. enterocolitica* (11.1%) [6]. *Yersinia enterocolitica* 2/O:5,27 was recently detected in bulk tank milk samples in Italy [19], highlighting the risk associated with consumption of raw or improperly pasteurised milk. In France, cattle, sheep and goats were identified as the main reservoir of *Y. enterocolitica* lineages 2/3–9b [20]. The present outbreak demonstrates that there is a risk of transmission of *Y. enterocolitica* from unpasteurised cheese.

In the French food industry, testing for *Yersinia* is not compulsory. When informed about this outbreak, the manufacturer included *Y. enterocolitica* in the panel of pathogenic bacteria to be tested: eleven samples were taken and tested negative by the microbiological laboratory of the cheese manufacturer. The method used by this laboratory was foodproof *Yersinia* Detection Kit (BIOTECON, Potsdam, Germany). In contrast, the analyses carried out by the food safety authorities, using ISO 10273:2017, yielded several positive results. The advantage of the foodproof *Yersinia* detection kit is the rapidity of results. However, the low sensitivity of this kit for detection of *Y. enterocolitica* from cheese in this outbreak highlights the need for a reliable and rapid method to detect pathogenic *Y. enterocolitica* in the food industry, and to provide isolates for tracing the source of contamination through genomic comparison.

Interestingly, during the site inspection on Farm A, we were informed that a suspicion of brucellosis was reported in early 2024 in the herd, based on positive serology. The suspicion was later ruled out. As there is a serological cross-reactivity between *Brucella* and *Y. enterocolitica* serotype O:9 [21], the seropositive results might have indicated presence of *Y. enterocolitica* in the herd. Noteworthy, when the outbreak was investigated, two suspected human cases of brucellosis were notified, with serum samples taken in March and June, respectively, and with a comment of consumption of this unpasteurised soft goat cheese (no brand detailed) before symptom onset. *Brucella* was ruled out as a cause of these infections by the National Reference Laboratory of *Brucella*. We requested testing of the sera for *Y. enterocolitica* infection; these patients had high IgG antibody titres (> 100 relative unit/mL) of *Yersinia enterocolitica*, leading to the retrospective identification of two cases with a probable link to the outbreak.

The identification of cross-border cases was facilitated by the Epipulse platform. As the use of genomic surveillance is limited to few countries, the number of cross-border cases is probably under-estimated.

## Conclusion

The identification of unpasteurised goat’s milk cheese as the source of a large outbreak of *Y. enterocolitica* 2/O:9 infections in humans emphasise the risks associated with unpasteurised dairy products. Several recommendations could be considered. Due to cross-reactivity, seropositive results for *Brucella* spp. may indicate presence of *Y. enterocolitica* in a herd. If *Brucella* infection is not confirmed, testing could be followed by analysis for *Yersinia*, as faecal contamination of milk may pose a serious health risk of unpasteurised dairy products. Goats' udders washing is not a mandatory practice in France. If unpasteurised milk is used for production of cheese without other heating steps, strict application of good hygiene practices, including udder washing, could decrease the risk of contamination of milk. This outbreak highlights the importance of understanding how *Y. enterocolitica* contaminates and persists in the food chain, from goat farms to consumers.

## Data Availability

Genomic sequences of all isolates have been deposited in the the National Center for Biotechnology (NCBI) (https://www.ncbi.nlm.nih.gov/) database under the BioProject ID PRJNA1192190 and BioSamples numbers SAMN45100424–SAMN45100621.
